# Stage-dependent predictors of atrial fibrillation recurrence after catheter ablation: a proof-of-concept analysis

**DOI:** 10.1186/s12872-026-06407-2

**Published:** 2026-08-08

**Authors:** Nabeel M. Abdo, Ziting Xie, Hesham A. Alyamani, Saeed Ghaithan, Qiying Xie

**Affiliations:** 1 Department of Cardiology, Xiangya Hospital, Central South University, 87 Xiangya Road, Kaifu District, Changsha, 410008 Hunan China; 2 Department of Neurology, Xiangya Hospital, Central South University, 87 Xiangya Road, Kaifu District, Changsha, 410008 Hunan China; 3 Department of Cardiology, West China Hospital, Sichuan University, 37 Guoxue Alley, Wuhou District, Chengdu, 610041 Sichuan China; 4 National Clinical Research Center for Geriatric Disorders, Xiangya Hospital, Central South University, 87 Xiangya Road, Kaifu District, Changsha, 410008 Hunan China

**Keywords:** Atrial fibrillation, Catheter ablation, Recurrence prediction, Disease-stage, Predictor Lifecycle, Risk stratification

## Abstract

**Background:**

Risk models for atrial fibrillation (AF) recurrence after catheter ablation apply the same predictor structure to every patient. This overlooks the biological differences between early and advanced disease. We examined whether the clinical predictors of 12-month recurrence differ across four disease-stage phenotypes.

**Methods:**

This retrospective cohort study enrolled 419 consecutive patients who underwent first-time radiofrequency ablation at Xiangya Hospital, Central South University, between October 2021 and June 2024. The institutional protocol was pulmonary vein isolation (PVI) alone, and patients undergoing concurrent left atrial appendage occlusion were excluded. Patients were classified by AF type and duration into four groups: early paroxysmal (Phenotype 1, ≤ 12 months, *n* = 225), long-standing paroxysmal (Phenotype 2, > 12 months, *n* = 86), recent-onset persistent (Phenotype 3, ≤ 18 months, *n* = 62), and long-duration persistent AF (Phenotype 4, > 18 months, *n* = 46). We performed phenotype-specific logistic regression, predictor × phenotype interaction testing, Kaplan–Meier and restricted mean survival analysis, and ROC analysis.

**Results:**

Twelve-month recurrence occurred in 88 patients (21.0%) and differed significantly across phenotypes (17.3%, 20.9%, 16.1%, and 45.7% in P1–P4; *p* < 0.001). Recurrence-free survival was worst in P4 (log-rank *p* < 0.001). In the overall model (AUC 0.672), the independent predictors were persistent AF type, longer duration, and hypertension, the last associated with lower recurrence. Phenotype-specific models revealed distinct profiles, and the sex × phenotype interaction was formally significant (*p* = 0.0042). In P1, younger age was the only predictor (AUC 0.701). In P3, female sex and hypertension were apparent predictors (apparent AUC 0.862; recurrence 43.8% in women vs. 6.5% in men), although this estimate is subject to optimism given only 10 events. In P4, no clinical variable was associated with recurrence despite its 45.7% rate, though power was insufficient to exclude moderate associations. The protective direction of hypertension strengthened across P1–P3 before disappearing in P4.

**Conclusion:**

In this exploratory, single-center analysis, the clinical predictors of post-ablation AF recurrence appeared to shift across the four disease-stage phenotypes, with formal statistical support for sex. We propose the Predictor Lifecycle as a testable hypothesis for prospective, multicenter validation. These findings are hypothesis-generating and do not constitute a validated risk-stratification framework for clinical use.

**Supplementary Information:**

The online version contains supplementary material available at https://doi.org/10.1186/s12872-026-06407-2.

## Introduction

Atrial fibrillation (AF) is the most common sustained cardiac arrhythmia, affecting an estimated 60 million people worldwide [1]. Beyond its hemodynamic effects, AF substantially increases the risk of stroke, heart failure, and all-cause mortality, placing a growing burden on health systems [2, 3]. Catheter ablation with pulmonary vein isolation has become the preferred rhythm-control strategy for symptomatic patients. Across multiple randomized trials, it outperforms antiarrhythmic drugs in maintaining sinus rhythm [4–7]. The 2024 ESC guidelines endorse it as a first-line option, within a shared decision-making approach, to reduce symptoms, recurrence, and progression in patients with paroxysmal AF [8].

Even with high procedural success in well-selected patients, recurrence after ablation remains common. One-year rates are reported between 31% and 58%, depending on patient characteristics and AF subtype [9, 10]. Identifying high-risk patients before ablation is therefore important for counselling, patient selection, and planning post-ablation surveillance.

Several clinical risk scores address this need, including APPLE [11], MB-LATER [12], ATLAS [13], CHA₂DS₂-VASc [14] and DR-FLASH [15]. Yet they share a basic limitation: each is derived from and applied to mixed AF populations without accounting for disease stage. AF is not static. It progresses from a trigger-driven, autonomically sensitive arrhythmia in its early stages to a substrate-dominated, structurally entrenched disease as it persists [16, 17], and the mechanisms driving recurrence shift accordingly. A model that treats early paroxysmal and long-duration persistent AF as equivalent is unlikely to capture this heterogeneity. The modest discrimination of existing scores, with C-statistics typically between 0.55 and 0.67 in external validation, may partly reflect this [18].

There is already a hint in the literature that this is what happens. Huang et al. [19] found that the APPLE score discriminated reasonably well in patients ablated within six months of diagnosis (AUC 0.74), but its performance fell substantially in those ablated later (AUC 0.58). The same score, applied to the same outcome, worked differently depending on where patients sat in the disease course. That observation invites a more general question. If a fixed score performs unevenly across disease stages, is it because the predictors themselves carry different weight at different stages?

Machine-learning models offer one way around the limitations of fixed-weight scores, and they have been applied to AF outcome prediction with encouraging results [20, 21]. Their advantage is flexibility. Saglietto et al. showed that a random forest model outperformed the APPLE score within the same registry population (AUC 0.721 versus 0.557) [22], and deep-learning models trained on multimodal data have reported still higher discrimination [20]. But this flexibility comes at a cost. These models can represent stage-dependent structure implicitly without ever revealing it, leaving the clinician with a number rather than an explanation. Our aim is different. We ask whether such structure exists in a form that can be stated plainly.

Two specific questions motivated this analysis. First, sex differences in AF outcomes are well recognized [23, 24], and women have been reported to carry a substantially higher recurrence risk after ablation [25]. Yet this difference attenuates once left atrial size and comorbidity burden are taken into account. This has led some authors to suggest that the apparent sex effect may reflect disease stage rather than biology [23]. If so, the effect should be visible at some stages and absent at others, a prediction that has not been directly tested. Second, it has not been systematically examined whether established risk factors such as hypertension retain predictive value across all stages, or fade as atrial remodeling advances.

We therefore propose the Predictor Lifecycle hypothesis: that the clinical predictors of AF recurrence change across disease-stage phenotypes, so that no single fixed-weight score can perform well across the full spectrum of AF. We explored this hypothesis in a consecutive ablation cohort as a proof-of-concept analysis. We stress at the outset that these observations are exploratory and are intended to motivate the framework, not to validate it. Conclusive testing will require larger, prospective, multicenter cohorts with harmonized rhythm surveillance.

## Methods

### Study design and population

This single-center, retrospective cohort study was conducted at Xiangya Hospital, Central South University, a high-volume tertiary referral center. We screened consecutive patients who underwent first-time radiofrequency catheter ablation (RFCA) with pulmonary vein isolation (PVI) for AF between October 2021 and June 2024.

Patients aged ≥ 18 years with a documented AF diagnosis were eligible if they had either a documented recurrence at any point after the blanking period, or at least 12 months of complete follow-up without recurrence. We excluded patients with incomplete records, loss to follow-up before 12 months in the absence of recurrence, ablation for non-AF arrhythmias, valvular AF, or concurrent left atrial appendage occlusion. The final cohort comprised 419 patients.

### Clinical phenotyping

Throughout, AF duration means time since first documented diagnosis, not the length of the current episode. The diagnosis date was taken from the earliest available medical record, referral note, or pre-procedural history. Where symptoms predated the first documented episode, the documented date was used. This may underestimate true duration in patients with prolonged undiagnosed AF, a misclassification more likely in P1 than in P3 or P4. AF burden was unavailable retrospectively and was not used.

**Fig. 1:**
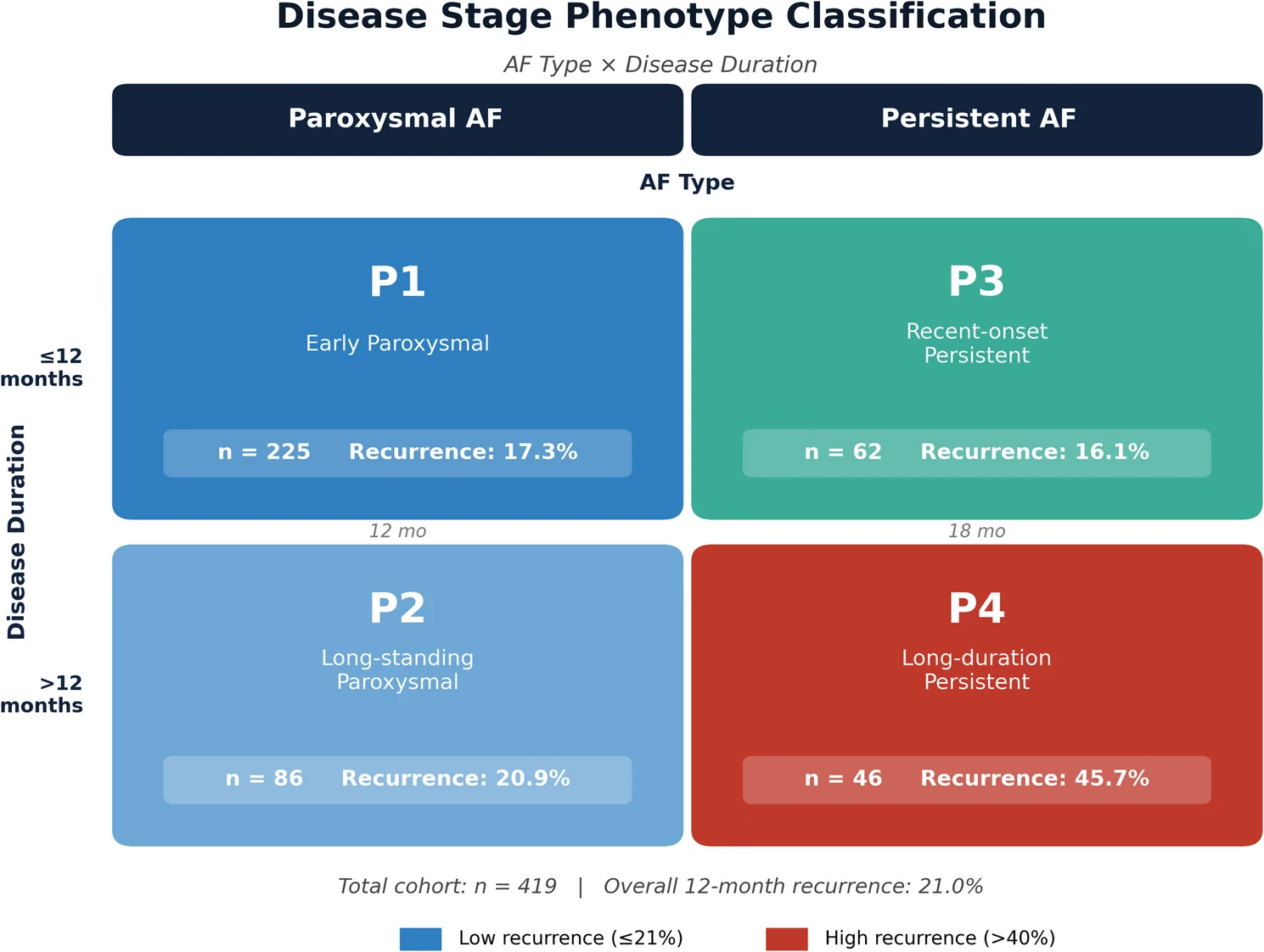
Disease stage phenotype classification framework. Patients were classified into four phenotypes based on AF type and disease duration. Recurrence rates represent 12-month AF recurrence after a 3-month blanking period. The 12-month paroxysmal threshold follows clinical convention and was retained on clinical grounds despite not being the outcome-maximizing cut-point in sensitivity analyses; the 18-month persistent threshold was data-derived, and repeating the full primary analysis under a pre-specified 12-month persistent split left all conclusions unchanged (Additional file, Table S5)

AF was classified per the 2024 ESC guidelines [8]. Four phenotypes were defined a priori by AF type and duration, the latter a proxy for atrial remodeling (Fig. 1): P1, early paroxysmal (≤ 12 months, *n* = 225); P2, long-standing paroxysmal (> 12 months, *n* = 86); P3, recent-onset persistent (≤ 18 months, *n* = 62); P4, long-duration persistent (> 18 months, *n* = 46). Sensitivity analyses across candidate cut-points (6–48 months paroxysmal; 12–36 months persistent) supported these thresholds. The 12-month paroxysmal cut-point gave stable discrimination throughout (χ² 19.6–20.9), and 18 months gave the strongest persistent separation (χ²=19.560, Cramér’s V = 0.216) over the 24-month alternative (χ²=11.892, V = 0.168) (Additional file, Table S1).

Heart failure was defined as a clinical diagnosis recorded by the treating physician, without retrospective re-adjudication or application of the HFA-PEFF score. Of 103 such patients, 71 (68.9%) had an ejection fraction ≥ 50%, which indicates a predominantly HFpEF phenotype. This classification heterogeneity is noted in the limitations.

### Data collection and follow-up

Baseline data came from pre-ablation records: demographics, AF characteristics, comorbidities, echocardiographic parameters, CHA₂DS₂-VASc score, and laboratory biomarkers. Hypertension was recorded as a binary diagnosis. Because blood pressure values and drug class were not systematically available, any protective association likely reflects treated rather than untreated disease.

All procedures followed the institutional protocol, which was PVI alone. Additional lesion sets were not routine practice here, consistent with trial evidence showing no incremental benefit over PVI. Intraoperative records were not systematically archived, so the lesion set could not be verified case by case, and uniformity rests on protocol rather than individual documentation. Forty-nine screened patients underwent concurrent left atrial appendage occlusion, which is separately documented, and were excluded. No screened patient was documented as undergoing ablation beyond PVI. All patients received antiarrhythmic therapy during the 3-month blanking period (amiodarone, dronedarone, or propafenone, at the electrophysiologist’s discretion).

Follow-up used all available records up to data extraction. The surveillance protocol comprised outpatient visits with 12-lead ECG at 1, 3, 6, and 12 months, with additional ECG, Holter, or event-monitor assessment whenever interval symptoms suggested arrhythmia. Every recurrence required objective documentation, and none was ascertained by symptoms alone. Antihypertensive exposure was defined as drug class on the post-ablation discharge list. Pre-existing versus newly prescribed status could not be distinguished retrospectively.

### Primary outcome

The primary outcome was AF recurrence within 12 months, defined a priori as any documented episode of atrial fibrillation, atrial flutter, or atrial tachycardia lasting ≥ 30 s after the 3-month blanking period, confirmed by ECG, Holter, or event recorder, per consensus reporting standards [26]. All 88 recurrences in this cohort were recorded as atrial fibrillation, with no flutter or tachycardia captured. Rhythm type was confirmed from the tracing where Holter monitoring was performed. Otherwise, recurrence was identified from readmission documentation stating that AF had recurred. Because rhythm type was not re-adjudicated in a structured, blinded manner across all 88 events, we cannot exclude that some readmission-documented events were flutter or tachycardia not distinguished from AF in the record. This limitation is discussed below.

### Secondary outcome

A secondary time-to-event outcome was time from ablation to first documented recurrence over all available follow-up, censored at data extraction or last contact. This was used only for the survival analyses. Logistic regression was restricted to the 12-month primary endpoint.

### Statistical analysis

Continuous variables were tested for normality (Shapiro–Wilk) and reported as mean ± SD with one-way ANOVA (Welch’s correction where variance was heterogeneous) or median [IQR] with Kruskal–Wallis test. Categorical variables are reported as n (%) with Pearson chi-square or Fisher’s exact test.

Recurrence-free survival was assessed by Kaplan–Meier analysis with log-rank testing. Because available follow-up was shorter in P4 than elsewhere, survival was compared using restricted mean survival time at common horizons of 24 and 36 months, with bootstrap confidence intervals (2,000 resamples). Logistic regression proceeded in two stages: univariate screening of all 21 candidate variables, then multivariable modeling by the Enter method. Variables entered if they showed univariate association at *p* < 0.10 and had prior clinical relevance. Those meeting the threshold but redundant with included variables were excluded, namely the CHA₂DS₂-VASc score, since its components were already entered. A minimum events-per-variable (EPV) of 10 was targeted overall. Phenotype-specific models were adapted to available events, with lower EPV acknowledged as a limitation (Additional file, Table S2). This yielded multivariable models in P1 (five variables, EPV 7.8), P3 (sex + hypertension, EPV 5.0), and P4 (four variables, EPV 5.0); univariate regression in P2; and Fisher’s exact test where complete separation occurred in P3. Current smoking met the univariate threshold (*p* = 0.091) but was not entered, since its apparent protective direction (OR 0.599) is biologically implausible and most likely reflects confounding or chance.

Discrimination was quantified by ROC AUC, with bootstrap confidence intervals for the P3 model. Given its EPV of 5.0, the P3 apparent AUC is subject to optimism and is reported as hypothesis-generating rather than validated. Calibration was assessed by the Hosmer–Lemeshow test for models with at least one confirmed predictor.

To test formally whether predictor effects differed by stage, we fitted predictor × phenotype interaction models in the pooled cohort for age, sex, and hypertension, assessing the overall interaction by likelihood-ratio test against the corresponding main-effects model. Given the few events in P3, the sex interaction was refitted using Firth’s penalized likelihood. As a further sensitivity analysis, we ran a parallel Cox proportional-hazards regression with time to first recurrence as the outcome, censored at last contact (Additional file, Table S3).

Post-hoc power calculations used the detectable-odds-ratio formula for logistic regression [27, 28], assuming a binary predictor at 50% prevalence, two-sided α of 0.05, 80% power, and the observed event proportion in each phenotype. The detectable odds ratio is the smallest true association each model had 80% power to detect. It is reported to make explicit what a null result can and cannot exclude.

Missing data were infrequent (< 5% for any variable) and handled by complete-case analysis within each model; no imputation was performed. During manuscript preparation the authors used Claude (Anthropic) for copy editing. The authors reviewed all output and take full responsibility for the article. Analyses used IBM SPSS Statistics v31.0 (IBM Corp., Armonk, NY) and Python 3.12, with two-tailed *p* < 0.05 considered significant.

## Results

### Study population and baseline characteristics

Of 491 patients screened, 72 were excluded, leaving 419 in the analytic cohort. The exclusions were 49 patients undergoing concurrent left atrial appendage occlusion, 12 undergoing repeat (non-first-time) ablation, and 11 lost to follow-up before 12 months. Among the 49 excluded for appendage occlusion, 24 had paroxysmal and 10 had persistent AF; AF type could not be determined from the available records for 15, and disease duration was not recoverable. The phenotype distribution was P1, *n* = 225 (53.7%); P2, *n* = 86 (20.5%); P3, *n* = 62 (14.8%); and P4, *n* = 46 (11.0%). Baseline characteristics are shown in Table 1.

**Table 1:** Baseline characteristics stratified by disease stage phenotype (*n* = 419)

Variable	P1 (*n* = 225)	P2 (*n* = 86)	P3 (*n* = 62)	P4 (*n* = 46)	Overall (*n* = 419)	*p*-value
Demographics						
Age (years)	58.8 ± 10.9	61.3 ± 10.4	61.1 ± 10.4	60.6 ± 11.0	59.9 ± 10.8	0.193
Sex — Male, n (%)	145 (64.4%)	50 (58.1%)	46 (74.2%)	32 (69.6%)	273 (65.2%)	0.209
BMI (kg/m²)	24.5 ± 3.4	25.0 ± 2.9	24.8 ± 3.3	25.1 ± 3.3	24.7 ± 3.3	0.602
Smoking — Current, n (%)	57 (25.3%)	19 (22.6%)	22 (35.5%)	7 (15.2%)	105 (25.2%)	0.102
Alcohol — Current, n (%)	52 (23.1%)	18 (21.4%)	17 (27.4%)	10 (21.7%)	97 (23.3%)	0.844
AF Characteristics						
AF Duration (months)‡	1.0 [0.5–6.0]	48.0 [24.0–72.0]	4.5 [2.0–8.0]	48.0 [24.0–117.0]	6.0 [1.0–24.0]	< 0.001†
Comorbidities						
Hypertension, n (%)	87 (38.7%)	44 (51.8%)	34 (54.8%)	24 (52.2%)	189 (45.2%)	0.035
Diabetes Mellitus, n (%)	39 (17.3%)	18 (20.9%)	8 (12.9%)	11 (23.9%)	76 (18.1%)	0.436
Heart Failure, n (%)	45 (20.0%)	16 (18.6%)	25 (40.3%)	17 (37.0%)	103 (24.6%)	0.001
Coronary Heart Disease, n (%)	85 (37.8%)	30 (34.9%)	17 (27.4%)	16 (34.8%)	148 (35.4%)	0.555
Prior Stroke/TIA, n (%)	20 (8.9%)	4 (4.7%)	8 (12.9%)	4 (8.7%)	36 (8.6%)	0.363
Vascular Disease, n (%)	60 (26.7%)	23 (26.7%)	15 (24.2%)	9 (19.6%)	107 (25.5%)	0.768
CHA₂DS₂-VASc Score‡	1.0 [1.0–3.0]	2.0 [1.0–3.0]	2.0 [1.0–3.0]	2.0 [1.0–3.0]	2.0 [1.0–3.0]	0.128
Cardiac Parameters						
Left Atrial Diameter (mm)§	39.8 ± 7.1	39.9 ± 5.7	45.2 ± 5.5	45.1 ± 6.5	41.2 ± 6.9	< 0.001
LVEF (%)‡	60.0 [55–65]	62.0 [55–66]	51.5 [42.2–59.0]	58.0 [53.0–63.0]	60.0 [53–65]	< 0.001
Biomarkers						
eGFR (mL/min/1.73 m²)	90.3 ± 18.6	91.0 ± 16.7	85.6 ± 21.4	85.1 ± 17.9	89.2 ± 18.7	0.104
NT-proBNP (pg/mL)‡¶	344 [109–733]	348 [100–1031]	936 [540–1813]	666 [314–1412]	452 [178–1041]	< 0.001
Uric Acid (µmol/L)	366.7 ± 97.4	372.7 ± 94.2	399.6 ± 96.8	387.4 ± 98.1	375.1 ± 97.2	0.093
LDL Cholesterol (mmol/L)‡	2.53 [2.06–3.19]	2.64 [2.17–3.16]	2.7 [2.2–3.2]	2.5 [2.1–2.8]	2.60 [2.11–3.16]	0.385
WBC (×10⁹/L)‡	6.0 [5.0–7.3]	6.3 [5.5–7.3]	6.2 [5.2–7.4]	6.0 [5.1–7.2]	6.2 [5.1–7.3]	0.840
Follow-up and Outcome						
Follow-up Duration (months)	27.9 ± 13.1	28.6 ± 13.1	28.8 ± 12.2	22.0 ± 13.5	27.5 ± 13.1	
12-Month AF Recurrence, n (%)	39 (17.3%)	18 (20.9%)	10 (16.1%)	21 (45.7%)	88 (21.0%)	< 0.001

Data were complete for all variables except: NT-proBNP (401/419), BMI (412/419), LVEF (413/419), LDL (414/419), left atrial diameter (415/419), smoking and alcohol status (417/419 each), and hypertension, coronary heart disease and WBC (418/419 each). Percentages and summary statistics are calculated on available data; all missingness was below 5% and was handled by complete-case analysis, as described in the Methods. Normally distributed variables compared by one-way ANOVA; homogeneity of variance was confirmed by Levene’s test for all such variables except left atrial diameter. Categorical variables compared by Pearson chi-square test. Follow-up duration is censored at recurrence and is therefore a function of the outcome rather than a baseline characteristic; no between-group test is reported

Abbreviations: *AF *atrial fibrillation, *BMI *body mass index, *CHA₂DS₂-VASc* congestive heart failure, hypertension, age ≥ 75, diabetes, stroke/TIA, vascular disease, age 65–74, female sex, *eGFR* estimated glomerular filtration rate, *IQR* interquartile range, *LDL* low-density lipoprotein, *LVEF* left ventricular ejection fraction, *NT-proBNP* N-terminal pro-B-type natriuretic peptide, *TIA* transient ischemic attack, *WBC* white blood cell count

† AF type and duration are phenotype-defining variables; p-values presented for descriptive purposes

‡ Median [IQR]; Kruskal–Wallis test. § Levene’s test violated (*p* = 0.011); Welch’s F confirmed (*p* < 0.001)

Demographics did not differ across phenotypes (all *p* > 0.05), but disease-severity markers showed a coherent gradient: left atrial diameter rose progressively from P1 to P4 (39.8 ± 7.1 to 45.1 ± 6.5 mm; *p* < 0.001), NT-proBNP was higher and LVEF lower in the persistent phenotypes (both *p* < 0.001), and heart failure prevalence stepped up at the paroxysmal-to-persistent transition (20.0% in P1 vs. 40.3% in P3; *p* = 0.001). Hypertension prevalence rose from 38.7% in P1 to 54.8% in P3 (*p* = 0.035).

Heart failure nonetheless showed no association with recurrence: 19.4% versus 21.5% without (*p* = 0.752; univariate OR 0.879, 95% CI 0.503–1.534, *p* = 0.649). Restricting analysis to patients without heart failure (*n* = 316, 68 events) preserved the cross-phenotype difference (20.0%, 21.4%, 13.5% and 41.4%; *p* = 0.038), including the P3–P4 step-change. The differential distribution therefore reflects the expected correlation between disease stage and comorbidity burden rather than confounding.

### Recurrence rates and survival

**Fig. 2:**
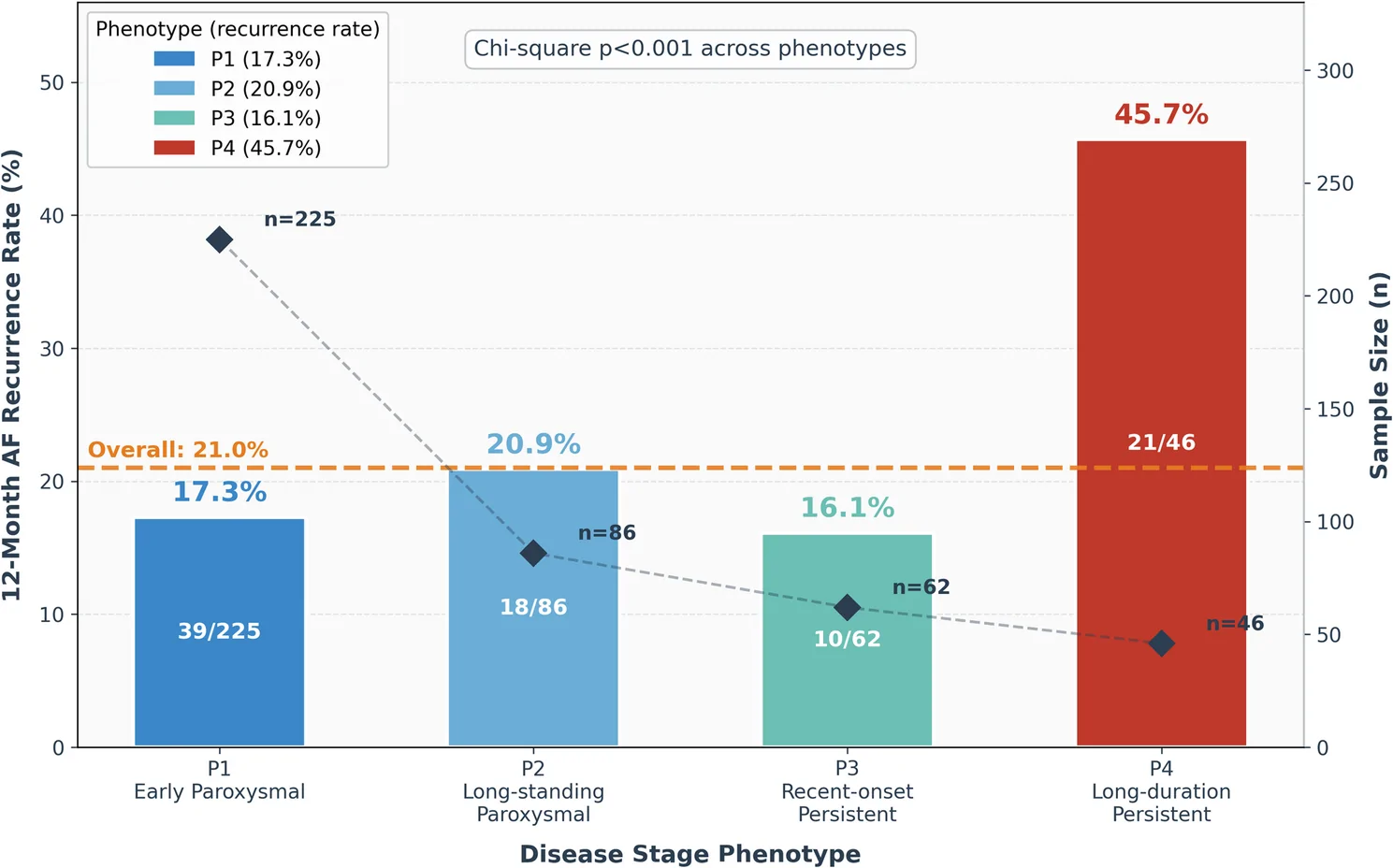
Recurrence rates and sample size by disease stage phenotype. Bars show 12-month AF recurrence rate (left y-axis); diamonds show sample size (right y-axis). Chi-square *p* < 0.001 across phenotypes. AF, atrial fibrillation

**Fig. 3:**
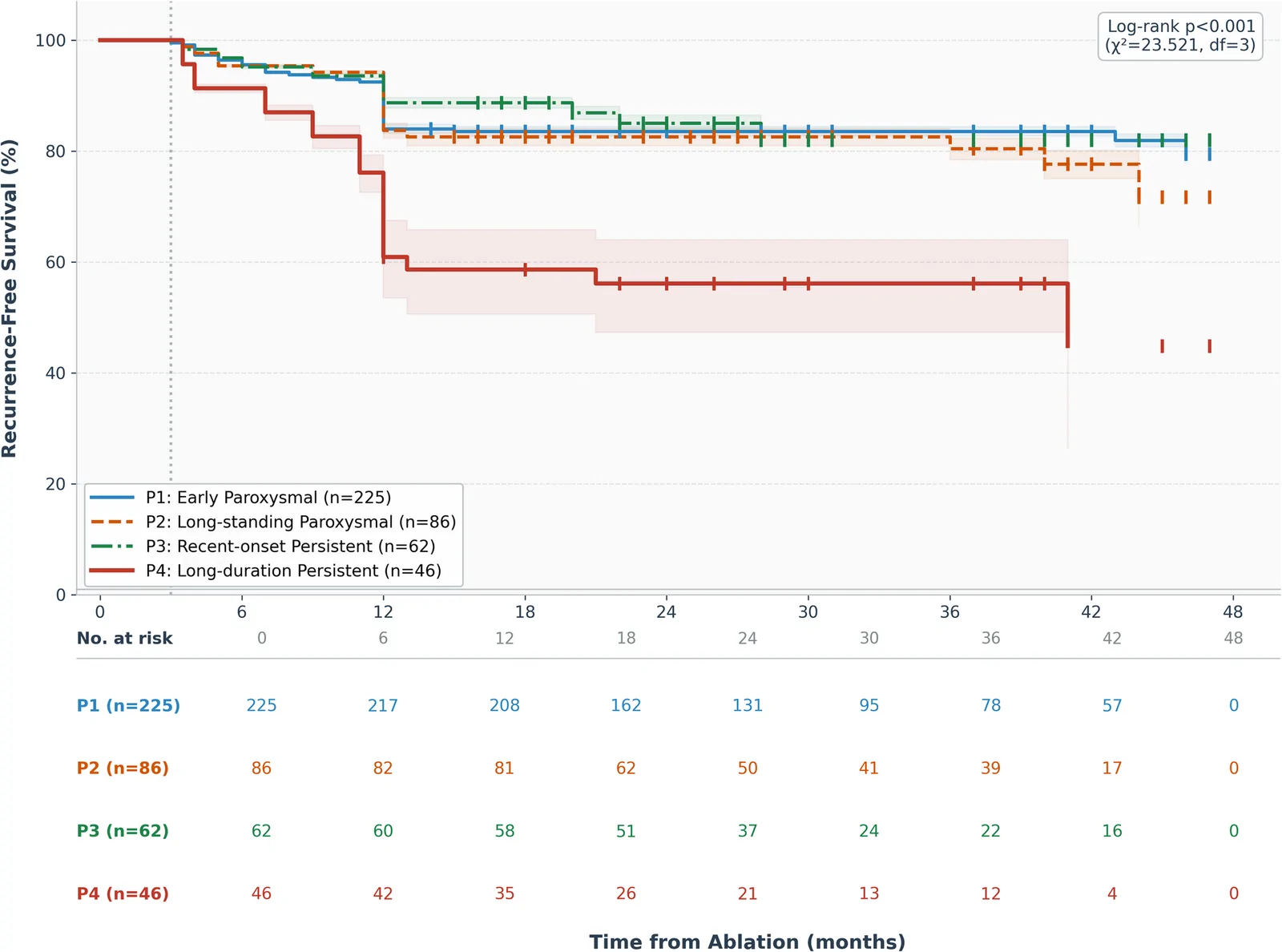
Kaplan–Meier recurrence-free survival curves stratified by disease stage phenotype. Phenotype 4 (long-duration persistent AF) shows significantly inferior recurrence-free survival compared with Phenotypes 1–3 from the third post-ablation month onwards (log-rank *p* < 0.001). The median survival time was not reached in Phenotypes 1, 2, or 3. Tick marks indicate censored observations. Numbers at risk are shown below the figure at 6-month intervals

Median follow-up was 26 months (IQR 17–41; range 3–47; mean 27.5 ± 13.1). All patients without recurrence had at least 12 months of follow-up by design. Those with recurrence were censored at the event, which accounts for the shorter follow-up in that subgroup. Twelve-month recurrence occurred in 88 of 419 patients (21.0%), with rates of 17.3%, 20.9%, 16.1% and 45.7% across P1–P4 (*p* < 0.001; Fig. 2). Recurrence-free survival was worst in P4 (log-rank *p* < 0.001; Fig. 3). Restricted mean survival time at 24 months was similar in P1, P2 and P3 (21.44, 21.22 and 21.95 months) but fell to 17.85 months in P4, a difference of − 3.60 months versus P1 (95% CI − 5.96 to − 1.33, *p* < 0.001), with the same pattern at 36 months (Table 2). This step-change, rather than a linear gradient, places the critical prognostic threshold at the transition to long-duration persistent disease. Median survival was not reached in P1–P3.

**Table 2:** Restricted mean survival time by disease-stage phenotype

Phenotype	*n*	Events	RMST 24 mo (95% CI)	Difference vs. P1 (95% CI)	*p*
P1 Early Paroxysmal	225	45	21.44 (20.70–22.16)	reference	—
P2 Long-standing Paroxysmal	86	24	21.22 (19.94–22.32)	−0.23 (− 1.66 to + 1.04)	0.717
P3 Recent-onset Persistent	62	13	21.95 (20.63–23.13)	+ 0.50 (− 1.02 to + 1.87)	0.495
P4 Long-duration Persistent	46	22	17.85 (15.63–20.02)	−3.60 (− 5.96 to − 1.33)	< 0.001

Phenotype			RMST 36 mo (95% CI)	Difference vs. P1 (95% CI)	*p*
P1 Early Paroxysmal			31.11 (29.76–32.35)	reference	—
P2 Long-standing Paroxysmal			30.15 (27.87–32.27)	−0.96 (−3.51 to +1.54)	0.488
P3 Recent-onset Persistent			31.30 (28.83–33.51)	+0.19 (−2.48 to +2.69)	0.887
P4 Long-duration Persistent			24.26 (20.44–28.10)	−6.85 (−10.84 to −2.78)	< 0.001

*RMST* restricted mean survival time

Estimated from Kaplan–Meier curves at common horizons of 24 and 36 months so that all phenotypes are compared over the same follow-up window. Confidence intervals and between-group p-values derived from 2,000 bootstrap resamples. Events are recurrences over total available follow-up. Values in months

Because the 18-month persistent threshold was selected on the outcome, we repeated the full primary analysis using a pre-specified 12-month split, matching the paroxysmal division. One patient reclassified and event counts were unchanged: recurrence was 17.3%, 20.9%, 16.4% and 44.7% (χ²=18.490, *p* = 0.0004), and every phenotype-specific finding held, including the sex × phenotype interaction (*p* = 0.0045) (Additional file, Table S5). Conclusions are therefore not threshold-dependent. Bootstrap analysis (2,000 resamples) confirmed 18 months as the modal maximizing threshold (54.7% of resamples versus 30.7% for 12 months; Additional file, Figure S1). The 12-month paroxysmal threshold was not outcome-maximizing, since 18, 24 and 48 months all yielded higher χ² (Additional file, Table S1). It was retained on clinical grounds.

### Overall multivariable logistic regression

Univariate analysis identified five variables associated with recurrence at *p* < 0.05: age (OR 0.970, *p* = 0.007), eGFR (OR 1.018, *p* = 0.012), AF type (OR 1.794, *p* = 0.024), hypertension (OR 0.522, *p* = 0.010) and vascular disease (OR 0.534, *p* = 0.042). AF duration approached significance (OR 1.004, *p* = 0.059) (Additional file, Table S2). In the six-variable model (Table 3), three predictors remained independent: persistent AF type (OR 1.968, 95% CI 1.152–3.364, *p* = 0.013), longer duration (OR 1.005, 95% CI 1.000–1.010, *p* = 0.038) and hypertension (OR 0.556, 95% CI 0.325–0.952, *p* = 0.032). Age, eGFR and vascular disease did not. The model achieved an AUC of 0.672 (95% CI 0.610–0.734, *p* < 0.001) with poor sensitivity at the 0.5 cut-off (3.4%), reflecting the limits of aggregate modeling in a heterogeneous population.

**Table 3:** Multivariable logistic regression — independent predictors of 12-month AF recurrence (overall cohort, *n* = 419)

Variable	OR	95% CI	*p*-value
Age (years)	0.986	0.960–1.012	0.285
eGFR (mL/min/1.73 m²)	1.012	0.997–1.028	0.122
AF Type (persistent vs. paroxysmal)	1.968*	1.152–3.364	0.013
AF Duration (months)	1.005*	1.000–1.010	0.038
Hypertension	0.556*	0.325–0.952	0.032
Vascular Disease	0.687	0.362–1.305	0.251

Model entered six variables simultaneously (Enter method). Overall AUC 0.672 (95% CI 0.610–0.734)

*OR *odds ratio, *CI *confidence interval, *AF *atrial fibrillation, *eGFR *estimated glomerular filtration rate

* Independent predictor (*p* < 0.05)

### Phenotype-specific Predictor Lifecycle analysis

Results are summarized in Table 4. In P1 (*n* = 225, 39 events), younger age was the sole independent predictor (adjusted OR 0.960, 95% CI 0.922–0.999, *p* = 0.046, a 4% reduction in odds per year). eGFR, heart failure, hypertension and vascular disease did not retain significance. The model reached an AUC of 0.701 (95% CI 0.616–0.785, *p* < 0.001).

**Table 4:** Phenotype-specific predictor analysis summary (Predictor Lifecycle)

Variable	P1 (*n* = 225)	P2 (*n* = 86)	P3 (*n* = 62)	P4 (*n* = 46)
Logistic Regression				
Age (years)	0.960* (0.922–0.999) *p* = 0.046	NS	NS	—
eGFR (mL/min/1.73 m²)	NS	NS	1.040† *p* = 0.058	—
AF Duration (months)	NS	NS	1.069† *p* = 0.055	—
Hypertension	NS	0.382† *p* = 0.084	0.156* *p* = 0.027	NS *p* = 0.489
Fisher's exact test — P3 variables with separation; P4 descriptive				
Sex (Female vs. Male)	—	—	43.8% vs. 6.5% *p* = 0.002	NS *p* = 1.000
Non-drinker vs. Drinker	—	—	22.2% (10/45) vs. 0% (0/17), Fisher *p* = 0.0496	—
Non-smoker vs. Smoker	—	—	22.5% (9/40) vs. 4.5% (1/22), Fisher *p* = 0.082	—

P2: univariate only. P4: four-variable confirmatory logistic regression (age, sex, hypertension, LA diameter); all non-significant. OR with 95% CI for logistic regression; recurrence rates with Fisher’s exact p for categorical comparisons. In Phenotype 3, no woman smoked or consumed alcohol; both variables are therefore entirely nested within male sex and are not independent predictors. Within non-smokers, recurrence was 43.8% in women versus 8.3% in men; within men, smoking status was not meaningfully associated with recurrence (4.5% versus 8.3%). For alcohol, complete separation was present (0 events among 17 drinkers). These rows are reported for completeness and are interpreted as proxies for sex (see Results and Discussion)

*NS *not significant, — not applicable

† Borderline significance after adjustment

* Independent predictor (*p* < 0.05) in multivariable model

In P2 (*n* = 86, 18 events), no variable reached significance, but power was adequate only for odds ratios ≥ 4.42, so moderate predictors cannot be excluded and this null is inconclusive. Hypertension showed the strongest direction-consistent association (OR 0.382, 95% CI 0.128–1.138, *p* = 0.084).

In P3 (*n* = 62, 10 events), two predictors were significant on univariate analysis: male sex (OR 0.090, 95% CI 0.019–0.415, *p* = 0.002; recurrence 43.8% in women vs. 6.5% in men) and hypertension (OR 0.156, 95% CI 0.030–0.811, *p* = 0.027; 5.9% vs. 28.6%). Both held in a two-variable model (sex: adjusted OR 0.070, 95% CI 0.012–0.389, *p* = 0.002; hypertension: adjusted OR 0.115, 95% CI 0.018–0.753, *p* = 0.024) and under Firth’s penalized likelihood (sex: OR 0.088, 95% CI 0.018–0.440, *p* = 0.003; hypertension: OR 0.151, 95% CI 0.027–0.847, *p* = 0.032), indicating the signal is unlikely to reflect small-sample bias alone. No woman in P3 smoked or drank alcohol, so both variables are nested within male sex. Among non-smokers alone, recurrence remained higher in women than men (43.8% vs. 8.3%), whereas among men smoking made little difference (4.5% vs. 8.3%). We therefore treat sex as the more fundamental variable and smoking and alcohol as proxies for it. The apparent AUC was 0.862 (95% bootstrap CI 0.750–0.955), but with 10 events across two variables (EPV 5.0) this is almost certainly inflated by overfitting. The P3 profile is an exploratory signal warranting confirmation, not a deployable predictor structure.

In P4 (*n* = 46, 21 events, recurrence 45.7%), a four-variable model (age, sex, hypertension, left atrial diameter; EPV 5.0, based on the 20 events with complete left atrial diameter data) identified no independent predictor (age *p* = 0.918, sex *p* = 0.673, hypertension *p* = 0.489, LA diameter *p* = 0.188). Power was adequate only for odds ratios ≥ 5.25. Detecting moderate effects (OR 2.0–3.0) would have required 32–72 events, so moderate predictors cannot be excluded. Discrimination is not reported, as AUC from a model with no significant predictor is uninformative. The highest recurrence rate combined with no detectable clinical predictor is compatible with, though it does not prove, a substrate-dominated state in which structural changes drive outcomes. Because substrate markers (LGE-MRI fibrosis, low-voltage areas, electrophysiological mapping) were not measured, this reading remains a hypothesis.

Cox models paralleled the logistic results throughout (Additional file, Table S3), with the P1 age effect (HR 0.953 per year, 95% CI 0.928–0.979, *p* = 0.0005) and the P3 sex and hypertension effects (HR 0.122 and 0.195, both *p* < 0.05) retaining significance.

### Formal interaction testing

Because comparing significance within subgroups does not establish that an effect differs between them, we tested the predictor × phenotype interactions directly (Table 5). Sex showed a significant interaction (LR χ²(3)=13.20, p= 0.0042), driven by P3 (female × P3 OR 14.46, 95% CI 2.64–79.29, p= 0.0021). This held under Firth's penalized likelihood (penalized LR χ²(3)=14.75, p= 0.0020) and survived Bonferroni correction for three tests. Interactions for age (LR χ²(3)=6.06, p= 0.109) and hypertension (LR χ²(3)=3.82, p=0.281) were not significant, although the age × P4 term reached nominal significance (OR 1.067, 95% CI 1.002–1.136, p= 0.044). Stage-dependence is therefore formally supported for sex. The age and hypertension patterns remain descriptive.

**Table 5:** Predictor × phenotype interaction models in the pooled cohort

Predictor	Overall interaction LR χ²(3), *p*	Interaction term (ref = P1)	OR	95% CI	*p*
Female sex	13.20, *p* = 0.0042	Female × P2	0.60	0.15–2.31	0.454
		**Female × P3**	**14.46**	**2.64–79.29**	**0.0021**
		Female × P4	1.67	0.39–7.19	0.493
Age (per year)	6.06, *p* = 0.109	Age × P2	1.058	0.996–1.124	0.067
		Age × P3	1.010	0.939–1.087	0.781
		Age × P4	1.067	1.002–1.136	0.044
Hypertension	3.82, *p* = 0.281	HTN × P2	0.78	0.21–2.99	0.719
		HTN × P3	0.32	0.05–1.98	0.220
		HTN × P4	2.08	0.52–8.41	0.304

Binary logistic regression with predictor × phenotype interaction terms; P1 is the reference phenotype. The overall interaction was assessed by likelihood-ratio (LR) test against the corresponding main-effects model. Cohort *n* = 419 with 88 events; the hypertension model used *n* = 418 owing to one missing value. With Bonferroni correction for three tests (α = 0.0167) the sex × phenotype interaction remains significant. Because P3 contained only 10 events, the sex interaction was refitted using Firth’s penalized likelihood: penalized LR χ² (3)= 14.75, *p* = 0.0020; female × P3 OR 12.48 (95% CI 2.39–65.06), *p* = 0.0027

*OR *odds ratio, *CI *confidence interval, *HTN *hypertension

### Hypertension across the disease lifecycle

**Fig. 4:**
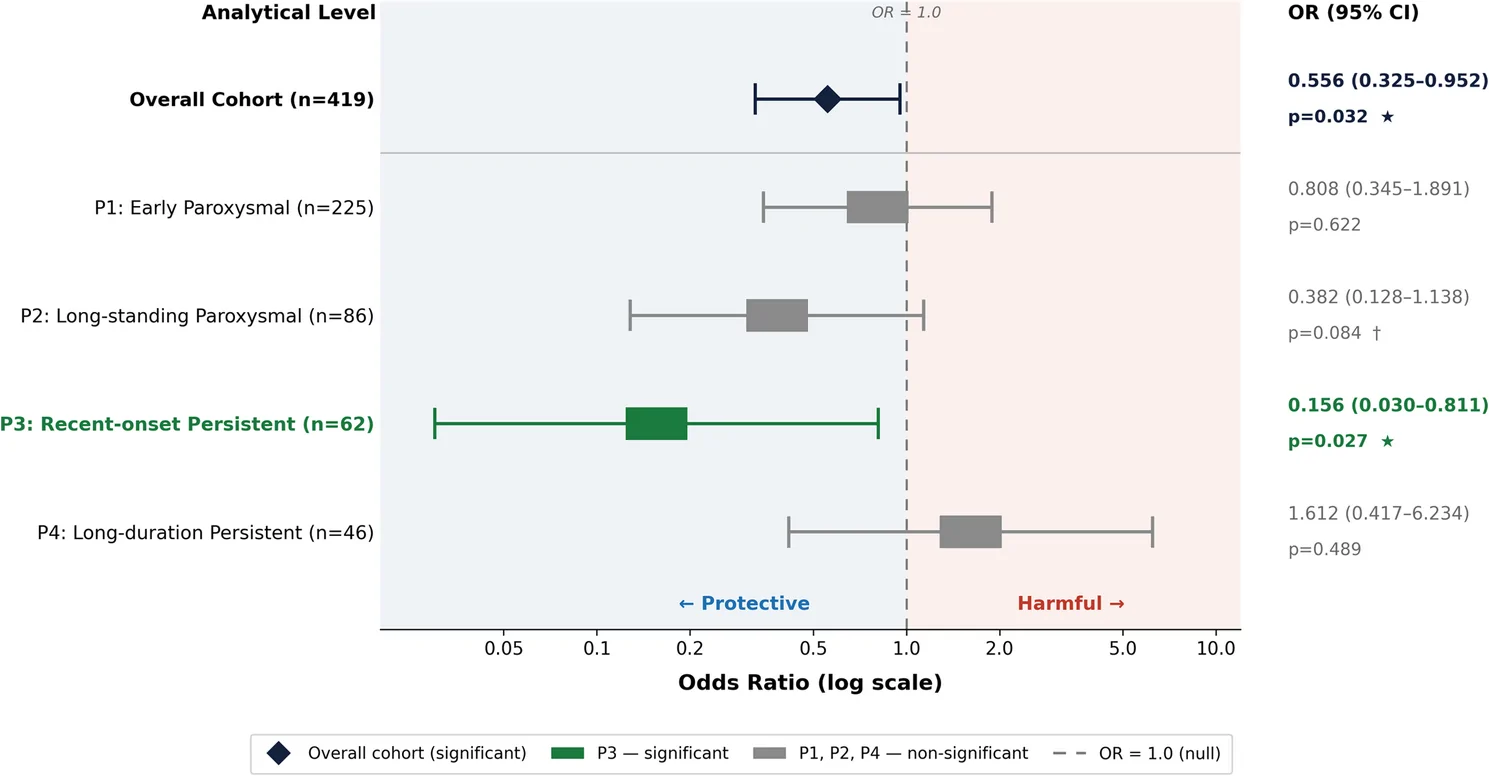
Forest plot: hypertension and AF recurrence across disease stages. Odds ratios with 95% confidence intervals are shown for each analytical level. The diamond represents the overall cohort estimate (adjusted, p=0.032); filled squares represent phenotype-specific estimates, with P3 (teal) reaching significance (p=0.027) and P1, P2 and P4 non-significant. Estimates are adjusted where a multivariable model was fitted (overall, P1, P4); the P2 and P3 values are univariate, as no multivariable model was fitted in P2. The vertical dashed line indicates OR=1.0. A protective direction (OR<1.0) is present in P1–P3, strengthening progressively across these phenotypes, and absent in P4. The formal hypertension × phenotype interaction test was not significant (LR χ²(3)=3.82, p=0.281), so this trajectory is descriptive rather than established. ★ significant at p < 0.05; † borderline significance. OR, odds ratio; CI, confidence interval; LR, likelihood ratio; AF, atrial fibrillation

Hypertension was independently associated with reduced recurrence overall (OR 0.556, 95% CI 0.325–0.952, *p* = 0.032). Across phenotypes, adjusted odds ratios were 0.808 (95% CI 0.345–1.891) in P1, 0.382 (95% CI 0.128–1.138) in P2, 0.156 (95% CI 0.030–0.811, *p* = 0.027) in P3, and 1.612 (95% CI 0.417–6.234, *p* = 0.489) in P4 (Fig. 4; Table 6). The apparent protective direction thus strengthened monotonically from P1 to P3 before disappearing in P4, though this trajectory warrants caution given the small within-phenotype event counts. 

**Table 6:** Hypertension association across disease stages — Predictor Lifecycle trajectory

Phenotype	OR (95% CI)	*p*-value	Significant?
Overall cohort (*n* = 419)	0.556 (0.325–0.952)	0.032	Yes ✓
P1: Early Paroxysmal (*n* = 225)	0.808 (0.345–1.891)	0.622	No
P2: Long-standing Paroxysmal (*n* = 86)	0.382 (0.128–1.138)	0.084	Borderline
P3: Recent-onset Persistent (*n* = 62)	0.156 (0.030–0.811)	0.027	Yes
P4: Long-duration Persistent (*n* = 46)	1.612 (0.417–6.234)	0.489	No

A protective direction (OR < 1.0) is present in P1–P3, strengthening progressively across these phenotypes, and absent in P4 (OR 1.612, p = 0.489). The formal hypertension × phenotype interaction test was not significant (LR χ²(3) = 3.82, p = 0.281), so this trajectory is descriptive rather than established

OR odds ratio, CI confidence interval

To test whether this reflected antihypertensive therapy rather than the diagnosis itself, we retrieved discharge medication data for all 189 patients with a hypertension diagnosis, coding four drug classes (RAAS inhibitors, beta-blockers, calcium channel blockers, diuretics). None was associated with recurrence: RAAS inhibitors (prevalence 72.0%) showed no protective association (16.2% recurrence on versus 13.2% off; OR 1.27, 95% CI 0.51–3.17, *p* = 0.61), and the others were similarly non-significant (all *p* > 0.6). Adding RAAS use to the overall model did not move the hypertension estimate toward the null (OR 0.556→0.436), and RAAS use itself was non-significant (OR 1.38, 95% CI 0.53–3.59, *p* = 0.51) (Additional file, Table S4). The apparent protective association is therefore not explained by documented drug class and most likely reflects residual confounding tied to the treated-hypertension label, or chance.

### Model calibration and discrimination

AUCs are reported as within-model descriptive summaries and are not compared across phenotypes, since discrimination depends on the case-mix of the population in which it is measured (Table 7). AUC was not estimated for P2 or P4, where no predictor was confirmed. All reported models calibrated acceptably by the Hosmer–Lemeshow test (overall χ²=9.047, df = 8, *p* = 0.338; P1 χ²=14.339, df = 8, *p* = 0.073; P3 χ²=0.781, df = 2, *p* = 0.677), with small-sample caution for P3. ROC curves are in Additional file, Figure S2.

**Table 7:** Model discrimination

Model	AUC	95% CI	*p*-value	H-L *p*
Overall cohort (*n* = 419, 6 variables)	0.672	0.610–0.734	< 0.001	0.338
P1: Early Paroxysmal AF (*n* = 225, 5 variables)	0.701	0.616–0.785	< 0.001	0.073
P3: Recent-onset Persistent AF (*n* = 62, 2 variables)	0.862	0.750–0.955	< 0.001	0.677

AUC reported for models with at least one confirmed independent predictor (*p* < 0.05): overall cohort and P1 (standard CI), P3 (bootstrap CI). AUC not reported for P2 (no significant predictors) or P4 (confirmatory null model). Bootstrap CI based on 2,000 resamples. AUC values are apparent estimates from derivation data without external validation

*AUC *area under the ROC curve, *CI *confidence interval

## Discussion

We interpret our findings within an explicit and deliberate scope. This proof-of-concept analysis was designed to test whether stage-specific predictor heterogeneity exists and is detectable within a single retrospective cohort, not to validate a clinical risk-stratification tool. Within that scope, the central finding is clear: the clinical predictors of AF recurrence differed across the four phenotypes, and for sex this difference was confirmed by formal interaction testing. Younger age was the only independent predictor in early paroxysmal AF. Female sex and hypertension emerged at the paroxysmal-to-persistent transition, where women had a sixfold higher recurrence rate than men. No clinical variable predicted recurrence in long-duration persistent AF. These observations are difficult to reconcile with the assumption underlying most published prediction models, that AF is a single disease amenable to a universal scoring system. We nonetheless note the boundaries of what a single-center cohort of 419 patients, with modest within-phenotype event counts (10–39 events) and no external validation, can establish. These findings motivate the Predictor Lifecycle framework as a hypothesis for prospective, multicenter testing rather than constituting proof of the concept.

### Age as a stage-specific predictor in early disease

That younger age independently predicted recurrence in P1 is counterintuitive but biologically coherent. Younger patients with early paroxysmal AF more often have autonomically mediated, trigger-driven arrhythmia with robust pulmonary vein firing that can persist despite technically successful isolation [29–31]. A multicenter prospective study of 2,799 patients found that those younger than 60 years had significantly higher 1-year recurrence (aHR 1.45, 95% CI 1.13–1.86) [32], though few studies have isolated the early-duration subgroup.

This association could, however, reflect differential detection: older patients may report fewer symptoms and have fewer documented recurrences, making younger age appear falsely as a risk factor. Such ascertainment bias cannot be resolved retrospectively. The P1 age effect should therefore be read as either a biological signal of trigger-driven recurrence in younger patients, or an artefact of age-related differences in symptom-driven detection. The present data cannot distinguish these.

### Sex at the paroxysmal-to-persistent transition

The association of female sex with recurrence specifically in P3, absent in every other phenotype and supported by a formal interaction test, is the most unexpected observation of this study. Women at this transition had a recurrence rate of 43.8% versus 6.5% in men (*p* = 0.002; adjusted OR 0.070, 95% CI 0.012–0.389). This finding speaks to an existing debate. Women have been reported to carry substantially higher post-ablation recurrence risk in pooled analyses [25], yet the difference attenuates once left atrial size and comorbidity burden are accounted for, leading some authors to argue that the apparent sex effect reflects disease stage rather than biology [23]. Our data offer a different reading of the same tension. Rather than sex being an artefact of stage, the sex effect may be real but stage-restricted, appearing at the paroxysmal-to-persistent transition and nowhere else. That would explain why pooled estimates are inconsistent, since averaging across stages would dilute an effect confined to one.

Mechanistically, this transition is a period of active atrial remodeling with upregulated inflammatory and fibrotic signaling. Sex-specific differences in this response, potentially mediated by estrogen’s influence on atrial electrophysiology and fibroblast signaling [33, 34], may create a more adverse remodeling environment in women at this particular stage. This warrants prospective validation with sex-stratified designs and biological sampling.

### Long-duration persistent AF and substrate saturation

In P4, a 45.7% recurrence rate coincided with the absence of any significant clinical predictor. One plausible reading is that, in advanced persistent AF, diffuse atrial fibrosis creates a substrate for re-initiation largely independent of the modifiable risk factors captured in routine assessment. However, we did not measure atrial substrate directly, and an alternative explanation cannot be excluded: that moderate predictors exist but went undetected given limited power (80% power only for OR ≥ 5.25). The substrate-dominance interpretation is thus a hypothesis motivated by, but not demonstrated by, these data [35].

For this group, imaging-based fibrosis quantification such as late gadolinium enhancement cardiac MRI [36], or electrophysiological mapping, may be needed for meaningful risk stratification. Beyond imaging, circulating biomarkers such as exosomal microRNAs are emerging as candidate indicators of atrial substrate [37, 38]. The step-change in restricted mean survival between P3 and P4, which was 21.95 months versus 17.85 at 24 months, and 31.30 versus 24.26 at 36 months, is consistent with the biological distinctiveness of this threshold. Unsupervised clustering studies point the same way: analyses of both clinical variables [39] and DECAAF II imaging data [40] have identified persistent-AF subgroups whose recurrence rates differ by 15–30% points, supporting the view that AF comprises biologically distinct subtypes rather than a single population.

### The hypertension trajectory

The cross-phenotype trajectory of hypertension illustrates what the Predictor Lifecycle concept would predict: an apparently protective association that strengthens from P1 to P3 and then disappears in P4. We emphasize that the protective direction is itself biologically counterintuitive and conflicts with meta-analytic evidence identifying hypertension as a risk factor for both incident AF and post-ablation recurrence [41]. The formal interaction test was not significant, so this trajectory is descriptive rather than established.

We initially suspected confounding by indication. Because hypertension was recorded as a binary label, patients carrying it are systematically those treated and in structured follow-up, while those untreated or undiagnosed are misclassified as non-hypertensive. The variable might therefore index treated hypertension and its drug exposure rather than elevated blood pressure itself. Prior evidence made this plausible: a meta-analysis of 15 studies found RAAS inhibitors reduced post-ablation recurrence (RR 0.83, 95% CI 0.70–0.98, *p* = 0.028) [42], whereas a study restricted to chronic persistent AF found no benefit from ACE inhibitors or ARBs [43]. Together these are compatible with stage-dependent attenuation of antihypertensive protection.

The drug-class analysis did not support this. No class was associated with recurrence, and adjustment for RAAS use did not move the hypertension estimate toward the null (Additional file, Table S4). The association therefore remains mechanistically unexplained and most plausibly reflects residual confounding tied to the treated-hypertension label, meaning greater healthcare engagement and rhythm surveillance, or chance from limited event counts. It is presented as an illustration of the kind of stage-dependent pattern the framework predicts, not as evidence of a mechanism. Prospective study incorporating blood pressure measurement, treatment characterization, and adherence data is needed.

### Model performance in context

The overall model achieved an AUC of 0.672, just below the threshold for acceptable clinical discrimination, while the P1 model reached 0.701. These values sit within the range reported for established AF ablation risk scores, which achieve 0.624–0.634 for APPLE across derivation and validation cohorts [11] and 0.627 for CHA₂DS₂-VASc in ablation outcome prediction, a performance its own investigators called mediocre [44]. The P3 model’s apparent AUC was 0.862. We report this value descriptively and do not rank it against published scores: it is uncorrected for optimism, derives from only 10 events, and was estimated in a different population from those in which the published scores were developed, so the comparison would not be meaningful. It should be treated as hypothesis-generating.

Independent evidence suggests score performance is itself stage-dependent. Huang et al. [19] found that APPLE discriminated reasonably in patients ablated within six months of diagnosis (AUC 0.74) but poorly in those ablated later (AUC 0.58), the same score and the same outcome performing differently according to where patients sat in the disease course. That a fixed-weight score should behave this way is exactly what the Predictor Lifecycle framework predicts, and it provides external support for the premise from a cohort other than our own.

### Implications if the hypothesis is confirmed

Should the Predictor Lifecycle hypothesis be confirmed in adequately powered prospective multicenter studies with harmonized surveillance, several implications would follow. Pre-procedural risk stratification might incorporate disease stage rather than a uniform score. In early paroxysmal AF, younger age, currently treated as low-risk in APPLE and similar scores, could become a readily available signal warranting closer surveillance. At the paroxysmal-to-persistent transition, a replicated sex effect could inform counselling and sex-stratified trial design. In long-duration persistent AF, where clinical variables appear uninformative, structural substrate imaging may provide the stratification that clinical scores cannot. If stage-dependence is confirmed, disease stage may be worth supplying to flexible models as an explicit stratifier rather than leaving them to infer it, which could improve interpretability without sacrificing performance. These implications are conditional on prospective confirmation and are not recommendations for current practice.

### Limitations

First, with 10–39 recurrence events per phenotype, and only 10 in P3 across a two-variable model (EPV 5.0), the phenotype-specific estimates are subject to small-sample bias and overfitting and should be regarded as exploratory signals rather than validated effect sizes.

Second, the four-phenotype framework is a typology, not a unidimensional staging system. It cross-classifies AF type and duration but imposes no strict severity ordering, and P2 and P3 are not unambiguously ordered on a single severity axis. Each patient was placed into one phenotype at baseline, and we compared outcomes between groups rather than following individuals as their AF progressed. The framework therefore shows how predictors differ across patients at different stages, not how one patient’s own risk factors change as their disease advances, which would require longitudinal follow-up this study was not designed for.

Third, the 49 patients excluded for concurrent left atrial appendage occlusion carry higher thromboembolic and bleeding risk than the analytic cohort, so this exclusion is a form of selection, although not one driven by intraoperative substrate assessment. Relative to the pools from which they were drawn, this represents exclusion of approximately 7.2% of paroxysmal and 8.5% of persistent patients. Lesion-set uniformity rests on institutional protocol rather than per-patient verification, as intraoperative records were not systematically archived.

Fourth, the duration thresholds were chosen for biological plausibility but assessed within the same dataset used for the predictor analyses, raising the possibility of cut-point overfitting. Bootstrap analysis confirmed the 18-month threshold as the modal choice but flagged the 12-month boundary as less stable, and repeating the full analysis under a pre-specified 12-month threshold left all conclusions unchanged. External validation of both remains essential.

Fifth, multiple candidate predictors were examined across four phenotypes without a pre-specified hierarchy or correction for multiple comparisons, increasing the risk of false positives. Although the framework and phenotypes were defined a priori, the predictors entered into each model were guided by univariate associations within the same dataset.

Sixth, the 12-month recurrence rate of 21.0% sits below published cohorts (typically 31–58%), and all 88 recurrences were recorded as AF with no atrial flutter or tachycardia, which is atypical for a PVI-only cohort, where atrial flutter alone occurred in 6.4% of the PVI-only arm of a large persistent-AF trial [45]. Surveillance relied on scheduled 12-lead ECG, with ambulatory monitoring only when symptoms prompted it, as is usual in routine care. Rhythm subtype was likewise recorded from clinical documentation rather than uniform tracing-level adjudication. Asymptomatic recurrence was therefore systematically under-detected, and the reported rates reflect clinically detected recurrence rather than true arrhythmia burden. This is the most plausible explanation both for the low overall rate and for the absence of recorded AFL/AT, which are frequently asymptomatic. Because under-detection applies across all four phenotypes, it affects absolute rates more than the comparisons between them, but it precludes direct comparison with trials using protocol-mandated monitoring. Only prospective study with systematic rhythm monitoring can resolve this.

Seventh, heart failure classification relied on documented diagnoses without re-adjudication, and 68.9% of cases had preserved ejection fraction. However, heart failure was not independently associated with recurrence (*p* = 0.752), and a sensitivity analysis excluding these patients (*n* = 316) preserved all key findings, indicating heart failure is a marker of advanced stage rather than an independent driver.

Finally, no external validation of the framework or its predictor profiles was performed; these findings are hypothesis-generating rather than practice-changing.

## Conclusion

In this exploratory, single-center proof-of-concept analysis, the clinical predictors of post-ablation AF recurrence appeared to differ across four disease-stage phenotypes: younger age in early paroxysmal AF, female sex and hypertension at the paroxysmal-to-persistent transition, and no significant clinical variable in long-duration persistent AF despite a 45.7% recurrence rate. Formal interaction testing confirmed stage-dependence for sex, while the age and hypertension patterns remained descriptive. We propose the Predictor Lifecycle as a hypothesis worthy of prospective testing: that the clinical predictors of AF recurrence shift across disease stages, so that uniform-weight models cannot capture this stage-dependence. These findings are hypothesis-generating. They do not constitute a validated risk-stratification framework and are not recommendations for current practice. Confirming or refuting the framework will require multicenter prospective cohorts with adequate per-phenotype event counts, harmonized rhythm surveillance, and external validation across diverse populations.

## Supplementary Information

Supplementary Material 1.

Supplementary Material 2.

## Data Availability

The data that support the findings of this study are available from the corresponding author upon reasonable request, subject to institutional data governance requirements.
