# Supplementary material for: Stage-dependent predictors of atrial fibrillation recurrence after catheter ablation: a proof-of-concept analysis
Source: BMC Cardiovasc Disord. 2026 Aug 8;26:1028. doi: 10.1186/s12872-026-06407-2 (PMC13733005; doi:10.1186/s12872-026-06407-2)
Supplement: Supplementary file 1 [file 12872_2026_6407_MOESM1_ESM.docx]

**Additional file**

Contents

[Additional file Table S1. Sensitivity Analysis of Disease Stage Phenotype Duration Thresholds	1](#_Toc232942242)

[Additional file Table S2: Univariate Logistic Regression and Variable Selection Logic — All Candidate Variables	3](#_Toc232942243)

[Additional file Table S3: Cox Proportional-Hazards Regression Results: Sensitivity Analysis	8](#_Toc232942244)

[Additional file Table S4: Antihypertensive Drug Class and 12-Month Recurrence Among Hypertensive Patients	11](#_Toc232942245)

[Additional file Figure S1	13](#_Toc232942246)

[Additional file Figure S2	13](#_Toc232942247)

# Additional file Table S1. Sensitivity Analysis of Disease Stage Phenotype Duration Thresholds

**Panel A: Paroxysmal AF Duration Threshold (persistent AF threshold fixed at 18 months)**

| **Threshold (months)** | **χ²** | **p-value** | **Cramér’s V** | **P1 n (rec %)** | **P2 n (rec %)** | **P3 n (rec %)** | **P4 n (rec %)** |
| --- | --- | --- | --- | --- | --- | --- | --- |
| 6 | 19.877 | <0.001 | 0.218 | 181 (16.6%) | 130 (20.8%) | 62 (16.1%) | 46 (45.7%) |
| **12** | **19.560** | **<0.001** | **0.216** | 225 (17.3%) | 86 (20.9%) | 62 (16.1%) | 46 (45.7%) |
| 18 | 20.204 | <0.001 | 0.220 | 231 (16.9%) | 80 (22.5%) | 62 (16.1%) | 46 (45.7%) |
| 24 | 19.918 | <0.001 | 0.218 | 254 (17.3%) | 57 (22.8%) | 62 (16.1%) | 46 (45.7%) |
| 36 | 19.555 | <0.001 | 0.216 | 266 (17.7%) | 45 (22.2%) | 62 (16.1%) | 46 (45.7%) |
| 48 | 20.855 | <0.001 | 0.223 | 278 (17.3%) | 33 (27.3%) | 62 (16.1%) | 46 (45.7%) |

Stratification performance was stable across all tested thresholds (χ² range 19.6–20.9, Cramér’s V 0.216–0.223). The 12-month threshold (highlighted) was selected as the clinically intuitive boundary between recent-onset and established paroxysmal AF. P3 and P4 distributions are unchanged as the paroxysmal threshold does not affect persistent AF classification.

**Panel B: Persistent AF Duration Threshold (paroxysmal AF threshold fixed at 12 months*)***

| **Threshold (months)** | **χ²** | **p-value** | **Cramér’s V** | **P1 n (rec %)** | **P2 n (rec %)** | **P3 n (rec %)** | **P4 n (rec %)** |
| --- | --- | --- | --- | --- | --- | --- | --- |
| 12 | 18.490 | <0.001 | 0.210 | 225 (17.3%) | 86 (20.9%) | 61 (16.4%) | 47 (44.7%) |
| **18** | **19.560** | **<0.001** | **0.216** | 225 (17.3%) | 86 (20.9%) | 62 (16.1%) | 46 (45.7%) |
| 24 | 11.892 | 0.008 | 0.168 | 225 (17.3%) | 86 (20.9%) | 76 (22.4%) | 32 (43.8%) |
| 30 | 11.892 | 0.008 | 0.168 | 225 (17.3%) | 86 (20.9%) | 76 (22.4%) | 32 (43.8%) |
| 36 | 11.972 | 0.008 | 0.169 | 225 (17.3%) | 86 (20.9%) | 82 (23.2%) | 26 (46.2%) |

The 18-month cutoff (highlighted) demonstrated the strongest stratification (χ² = 19.560, Cramér’s V = 0.216), with a substantial drop in discrimination at ≥24 months (χ² = 11.892). P1 and P2 distributions are unchanged as the persistent threshold does not affect paroxysmal AF classification.

**General note:** Four-group phenotype classification: P1, early paroxysmal AF (paroxysmal, duration ≤12 months); P2, long-standing paroxysmal AF (paroxysmal, >12 months); P3, recent-onset persistent AF (persistent, ≤18 months); P4, long-duration persistent AF (persistent, >18 months). Recurrence rates (rec %) represent 12-month AF recurrence after a 3-month blanking period. χ² from Pearson chi-square test across all four phenotypes. Both thresholds were derived from the analytic cohort and require external validation.

# Additional file Table S2: Univariate Logistic Regression and Variable Selection Logic — All Candidate Variables

Univariate logistic regression for all 21 candidate variables in the full cohort (n=419, 88 events). Variables were eligible for entry into the overall multivariable model if they met a liberal univariate threshold of p<0.10 AND were judged clinically relevant; variables meeting the threshold but clinically redundant with already-included variables were not separately entered. A minimum events-per-variable (EPV) ratio of 10 was targeted for the overall model (88 events ÷ 6 variables = EPV 14.7); phenotype-specific models were adapted to lower within-phenotype event counts, with resulting EPV constraints acknowledged as a study limitation.

| Variable | OR | 95% CI | p-value | Entered model | Rationale |
| --- | --- | --- | --- | --- | --- |
| Age (years) | 0.970 | 0.949–0.992 | 0.007 | Yes | Significant; clinically relevant |
| Sex | 0.979 | 0.598–1.602 | 0.932 | No | Did not meet threshold |
| eGFR (mL/min/1.73m²) | 1.018 | 1.004–1.032 | 0.012 | Yes | Significant; clinically relevant |
| Body Mass Index (kg/m²) | 0.983 | 0.914–1.057 | 0.637 | No | Did not meet threshold |
| Current Smoking | 0.599 | 0.331–1.085 | 0.091 | No | Near-significant, protective direction biologically implausible, not pre-specified |
| Current Alcohol Use | 0.619 | 0.337–1.138 | 0.123 | No | Did not meet threshold |
| AF Type (persistent vs paroxysmal) | 1.794 | 1.081–2.977 | 0.024 | Yes | Significant; core disease marker |
| AF Duration (months) | 1.004 | 1.000–1.009 | 0.059 | Yes | Near-significant; core disease marker |
| Heart Failure | 0.879 | 0.503–1.534 | 0.649 | No | Did not meet threshold |
| Coronary Heart Disease | 0.715 | 0.430–1.190 | 0.197 | No | Did not meet threshold |
| Hypertension | 0.522 | 0.319–0.856 | 0.010 | Yes | Significant; clinically relevant |
| Diabetes Mellitus | 1.210 | 0.671–2.184 | 0.526 | No | Did not meet threshold |
| Prior Stroke or TIA | 1.504 | 0.696–3.250 | 0.299 | No | Did not meet threshold |
| Vascular Disease | 0.534 | 0.291–0.978 | 0.042 | Yes | Significant; clinically relevant |
| CHA₂DS₂-VASc Score | 0.882 | 0.760–1.024 | 0.098 | No | Redundant (composite of included components) |
| LVEF (%) | 1.001 | 0.978–1.024 | 0.943 | No | Did not meet threshold |
| Left Atrial Diameter (mm) | 0.987 | 0.954–1.022 | 0.463 | No | Did not meet threshold |
| NT-proBNP (pg/mL) | 1.000 | 1.000–1.000 | 0.359 | No | Did not meet threshold |
| Uric Acid (μmol/L) | 1.001 | 0.999–1.004 | 0.338 | No | Did not meet threshold |
| LDL Cholesterol (mmol/L) | 0.902 | 0.660–1.231 | 0.515 | No | Did not meet threshold |
| White Blood Cell Count | 1.004 | 0.881–1.144 | 0.955 | No | Did not meet threshold |

**Footnote**: Six variables met both criteria and were entered into the overall multivariable model (EPV 14.7). Phenotype-specific models used reduced variable sets adapted to within-phenotype event counts: P1 (39 events), P2 (18 events), P3 (10 events; two-variable model, EPV 5.0), and P4 (20 events available for the four-variable model after exclusion of one case with missing left atrial diameter; EPV 5.0); sub-threshold EPV in P2–P4 is acknowledged in the Limitations. Shaded rows denote variables entered into the overall multivariable model. OR, odds ratio; CI, confidence interval; AF, atrial fibrillation; CHA₂DS₂-VASc, composite thromboembolic risk score; eGFR, estimated glomerular filtration rate; EPV, events-per-variable; LVEF, left ventricular ejection fraction; LDL, low-density lipoprotein; NT-proBNP, N-terminal pro-B-type natriuretic peptide; TIA, transient ischaemic attack.

# Additional file Table S3: Cox Proportional-Hazards Regression Results: Sensitivity Analysis

Cox proportional-hazards regression as a time-to-event sensitivity analysis. Time-to-first-recurrence was used as the outcome, with follow-up censored at last documented contact. Hazard ratios (HR) and 95% confidence intervals are reported. Bold entries denote statistically significant associations (p<0.05).

**Part A. Overall multivariable Cox model (n=419)**

| Predictor | HR | 95% CI | p-value |
| --- | --- | --- | --- |
| AF Type (persistent vs paroxysmal) | 1.851 | 1.187–2.887 | 0.007 |
| AF Duration (per month) | 1.004 | 1.000–1.007 | 0.028 |
| Hypertension | 0.611 | 0.382–0.978 | 0.039 |
| Age (per year) | 0.984 | 0.962–1.006 | 0.149 |
| eGFR (per mL/min/1.73 m²) | 1.009 | 0.995–1.022 | 0.194 |

**Part B. Phenotype-specific univariate Cox models**

| Phenotype | n | Events | Predictor | HR | 95% CI | p-value |
| --- | --- | --- | --- | --- | --- | --- |
| P1 (Early paroxysmal) | 225 | 39 | Age | 0.953 | 0.928–0.979 | 0.0005 |
|  |  |  | Sex (male) | 1.292 | 0.656–2.545 | 0.459 |
|  |  |  | Hypertension | 0.521 | 0.255–1.067 | 0.075 |
| P2 (Long-standing paroxysmal) | 86 | 18 | Age | 0.998 | 0.954–1.044 | 0.927 |
|  |  |  | Sex (male) | 1.932 | 0.691–5.399 | 0.209 |
|  |  |  | Hypertension | 0.451 | 0.170–1.198 | 0.110 |
| P3 (Recent-onset persistent) | **62** | 10 | Sex (male) | 0.122 | 0.032–0.472 | 0.002 |
|  |  |  | Hypertension | 0.195 | 0.042–0.903 | 0.037 |
|  |  |  | Age | 0.960 | 0.907–1.015 | 0.151 |
| P4 (Long-duration persistent) | 46 | 21 | Age | 1.000 | 0.961–1.040 | 0.986 |
|  |  |  | Sex (male) | 0.739 | 0.298–1.833 | 0.514 |
|  |  |  | Hypertension | 1.067 | 0.453–2.514 | 0.881 |

**Interpretation**: The Cox proportional-hazards model parallels the logistic regression analysis with the binary 12-month outcome, but uses time-to-first-recurrence with right-censoring. Key findings — younger age in P1 and female sex/hypertension in P3 — are reproduced under the time-to-event framework, with the P1 age effect reaching p=0.0005 in Cox regression versus p=0.046 in the binary logistic regression. P4 shows no statistically significant predictor under either framework. Univariate associations in P2 did not reach significance under either analytic approach. This convergence between logistic and Cox regression frameworks supports the robustness of the phenotype-specific predictor profiles to choose of outcome definition*.*

# Additional file Table S4: Antihypertensive Drug Class and 12-Month Recurrence Among Hypertensive Patients

Sensitivity analysis examining whether antihypertensive drug class explains the apparent protective association of hypertension with AF recurrence. Discharge medication data were retrieved for all 189 patients carrying a hypertension diagnosis. Each drug class was coded as a binary variable (present/absent). Univariate logistic regression was used to test the association of each class with 12-month recurrence.

**Panel A. Drug class associations among hypertensive patients (n=189)**

| **Drug class** | **Patients on drug, n (%)** | **Recurrence on drug** | **Recurrence off drug** | **OR (95% CI)** | **p-value** |
| --- | --- | --- | --- | --- | --- |
| RAAS inhibitor (ACEi/ARB) | 136 (72.0%) | 16.2% | 13.2% | 1.27 (0.51–3.17) | 0.612 |
| Beta-blocker | 60 (31.7%) | 16.7% | 14.7% | 1.16 (0.50–2.67) | 0.731 |
| Calcium channel blocker | 30 (15.9%) | 16.7% | 15.1% | 1.12 (0.39–3.23) | 0.827 |
| Diuretic | 35 (18.5%) | 14.3% | 15.6% | 0.90 (0.32–2.56) | 0.847 |

**Panel B. Effect of adjusting for RAAS inhibitor use on the hypertension estimate**

| Model | Hypertension OR (95% CI) | p-value |
| --- | --- | --- |
| Base multivariable model (without RAAS) | 0.556 (0.325–0.952) | 0.032 |
| Model additionally adjusted for RAAS use | 0.436 (0.176–1.084) | 0.074 |
| RAAS inhibitor use (in adjusted model) | 1.381 (0.532–3.588) | 0.507 |

**Interpretation**: No antihypertensive drug class was associated with 12-month recurrence among hypertensive patients (all p>0.6). RAAS inhibitor use, the most prevalent class (72.0%), showed no protective association and, if anything, trended toward higher recurrence (16.2% on RAAS vs 13.2% off RAAS). Adjusting the overall multivariable model for RAAS use did not attenuate the hypertension estimate toward the null (OR 0.556 → 0.436); RAAS use itself was non-significant (OR 1.38, p=0.51). These findings indicate that the apparent protective association of hypertension is not explained by documented antihypertensive drug class, and most likely reflects residual confounding associated with the treated-hypertension diagnostic label (e.g., greater healthcare engagement and rhythm surveillance) or chance arising from limited event counts, rather than a pharmacological effect.

# Table S5 threshold sensitivity

**Table S5. Full primary analysis under the data-derived 18-month and the pre-specified 12-month persistent thresholds.**

| **Analysis** | **18-month (primary)** | **12-month (pre-specified)** |
| --- | --- | --- |
| Recurrence P1–P4 | 17.3 / 20.9 / 16.1 / 45.7% | 17.3 / 20.9 / 16.4 / 44.7% |
| χ² (p); Cramér’s V | 19.560 (0.0002); 0.216 | 18.490 (0.0004); 0.210 |
| P3 / P4 group size | 62 / 46 | 61 / 47 |
| P1 age, adjusted OR | 0.960 (0.922–0.999), p=0.046 | 0.960 (0.922–0.999), p=0.046 |
| P2 hypertension, OR | 0.382 (0.128–1.138), p=0.084 | 0.382 (0.128–1.138), p=0.084 |
| P3 female sex, adjusted OR | 14.38 (2.57–80.30), p=0.0024 | 14.24 (2.54–79.66), p=0.0025 |
| P3 hypertension, adjusted OR | 0.115 (0.018–0.753), p=0.024 | 0.117 (0.018–0.765), p=0.025 |
| P4 predictors | none significant (all p>0.18) | none significant (all p>0.20) |
| Sex × phenotype interaction | LR χ²(3)=13.20, p=0.0042 | LR χ²(3)=13.05, p=0.0045 |
| Age × phenotype interaction | LR χ²(3)=6.06, p=0.109 | LR χ²(3)=5.88, p=0.118 |
| Hypertension × phenotype | LR χ²(3)=3.82, p=0.281 | LR χ²(3)=3.41, p=0.333 |

*Reclassification under the 12-month threshold affected a single patient (AF duration 14 months, no recurrence), who moved from P3 to P4; event counts within phenotypes were unchanged. All phenotype-specific conclusions and all interaction results were preserved. OR, odds ratio; LR, likelihood ratio.*

# Additional file Figure S1

Bootstrap stability analysis of the 18-month duration threshold separating recent-onset (P3) from long-duration (P4) persistent atrial fibrillation. In each of 2,000 bootstrap resamples of the persistent AF cohort, the χ² statistic for the association between duration dichotomized at each candidate threshold (12, 18, 24, 30, and 36 months) and 12-month recurrence was computed, and the threshold maximizing χ² was recorded. Bars show the percentage of resamples in which each candidate threshold was selected as optimal. The 18-month threshold was the modal best estimate (selected in 54.7% of resamples), followed by the 12-month threshold (30.7%); the 24-, 30-, and 36-month thresholds were rarely selected (5.2%, 0.0%, and 9.4%, respectively). This supports the 18-month threshold as the most stable data-driven choice while indicating residual uncertainty, particularly at the 12-month boundary. No patient had AF duration between 24 and 30 months, so these two thresholds produce identical partitions of the persistent cohort; ties were resolved to the lower value, which is why 30 months is never selected as the maximizing threshold.

# Additional file Figure S2

Receiver operating characteristic (ROC) curves for the overall and phenotype-specific multivariable logistic regression models. The overall model (six predictors) achieved an area under the curve (AUC) of 0.672 (95% CI 0.610–0.734); the P1 model (early paroxysmal) an AUC of 0.701 (95% CI 0.616–0.785); and the P3 two-variable model (sex and hypertension) an apparent AUC of 0.862 (95% bootstrap CI 0.750–0.955). The P3 estimate is flagged as apparent and subject to optimism, as it derives from only 10 events (events-per-variable 5.0) and is therefore inflated by overfitting; it should not be interpreted as a validated performance estimate. ROC curves are not displayed for P2 and P4, as no independent predictor was identified within either phenotype. Curves are presented separately by model and are not superimposed, as discrimination depends on the case-mix of the population in which it is measured and is therefore not comparable across phenotypes. AUC, area under the curve; CI, confidence interval; P1–P4, disease-stage phenotypes 1–4.
